# Role of RNase H1 in DNA repair: removal of single ribonucleotide misincorporated into DNA in collaboration with RNase H2

**DOI:** 10.1038/srep09969

**Published:** 2015-05-07

**Authors:** Elias Tannous, Eiko Kanaya, Shigenori Kanaya

**Affiliations:** 1 Department of Material and Life Science, Graduate School of Engineering, Osaka University, 2-1 Yamadaoka, Suita, Osaka 565-0871, Japan

## Abstract

Several RNases H1 cleave the RNA-DNA junction of Okazaki fragment-like RNA-DNA/DNA substrate. This activity, termed 3’-junction ribonuclease (3’-JRNase) activity, is different from the 5’-JRNase activity of RNase H2 that cleaves the 5’-side of the ribonucleotide of the RNA-DNA junction and is required to initiate the ribonucleotide excision repair pathway. To examine whether RNase H1 exhibits 3’-JRNase activity for dsDNA containing a single ribonucleotide and can remove this ribonucleotide in collaboration with RNase H2, cleavage of a DNA_8_-RNA_1_-DNA_9_/DNA_18_ substrate with *E. coli* RNase H1 and H2 was analyzed. This substrate was cleaved by *E. coli* RNase H1 at the (5’)RNA-DNA(3’) junction, regardless of whether it was cleaved by *E. coli* RNase H2 at the (5’)DNA-RNA(3’) junction in advance or not. Likewise, this substrate was cleaved by *E. coli* RNase H2 at the (5’)DNA-RNA(3’) junction, regardless of whether it was cleaved by *E. coli* RNase H1 at the (5’)RNA-DNA(3’) junction in advance or not. When this substrate was cleaved by a mixture of *E. coli* RNases H1 and H2, the ribonucleotide was removed from the substrate. We propose that RNase H1 is involved in the excision of single ribonucleotides misincorporated into DNA in collaboration with RNase H2.

Ribonuclease H (RNase H) is an endoribonuclease that specifically cleaves the RNA strand of RNA/DNA hybrids^1^. It cleaves the PO-3’ bond of the substrate with a two-metal-ion catalysis mechanism, in which two divalent cations, such as Mg^2+^ and Mn^2+^, directly participate in the catalytic function^2^. RNase H is widely present in various organisms, including bacteria, archaea and eukaryotes. It is also present in retroviruses as a C-terminal domain of reverse transcriptase (RT). These RNases H are classified into type 1 and type 2 RNases H based on the difference in their amino acid sequences^3^,^4^. Type 1 RNases H include prokaryotic and eukaryotic RNases H1 and retroviral RNase H. Type 2 RNases H include prokaryotic and eukaryotic RNases H2 and bacterial RNase H3. These RNases H exist in a monomeric form^4^, except for eukaryotic RNases H2, which exist in a heterotrimeric form^5^,^6^.

Most organisms have multiple RNases H within a single cell^3^,^4^. They are mostly RNases H1 and H2 and occasionally RNases H2 and H3. Loss of these enzymes has shown different effect on various organisms. While loss of all functional RNases H renders *B. subtilis* unable to grow^7^, it leads to temperature sensitivity^7^ and the accumulation of untreated R loops, leading to DNA replication initiation at sites other than the oriC^8^, in *E. coli*, and higher sensitivity to alkylating agents in *Saccharomyces cerevisiae*^9^. In mouse, loss of either RNase H1^10^ or RNase H2^11^,^12^ causes embryonic lethality. In human, mutations in RNase H2 subunits cause Aicardi-Goutieres syndrome and mimic congenital viral brain infection^13^. In retroviruses, inactivation of the RNase H domain of RT abolishes virus infectivity^14^,^15^ and therefore retroviral RNase H is regarded as a target for AIDS therapy^16^,^17^.

RNase H2 is required to initiate the ribonucleotide excision repair (RER) pathway both in eukaryotes^11^,^12^,^18^,^19^ and prokaryotes^20^ because of its junction ribonucelase (JRNase) activity. This activity cleaves dsDNA containing a single rNMP (dsDNA^R1^) at the 5’-side of the ribonucleotide^21^,^22^,^23^,^24^. Subsequent removal of the ribonucleotide is catalyzed by several enzymes that all together form the RER pathway^11^,^18^,^20^. RNase H1 does not exhibit JRNase activity^21^,^25^,^26^,^27^,^28^. However, RNases H1 from *Halobacterium* sp. NRC-1 (Halo-RNase H1)^29^, *Sulfolobus tokodaii* (Sto-RNase H1)^30^, *Thermotoga maritima*^26^, and *E. coli*^26^,^31^ exhibit a weak activity that catalyzes the cleavage of an Okazaki fragment-like substrate at the RNA-DNA junction. To distinguish this activity from JRNase activity of RNase H2, that catalyzes the cleavage of an RNA-DNA/DNA substrate at the 5’-side of the ribonucleotide of the RNA-DNA junction^31^, this activity and JRNase activity of RNase H2 will be designated as 3’- and 5’-JRNase activities respectively hereafter (Fig. 1a,b). The RNA-DNA junction of an Okazaki fragment-like substrate containing a single ribonucleotide is not cleaved by Halo-RNase H1^29^ and Sto-RNase H1^30^, suggesting that an upstream duplex structure is necessary for recognition of this junction by RNase H1. However, it remains to be determined whether the RNA-DNA junction of this substrate is cleaved by RNase H1 by supplying a short RNA fragment that facilitates the formation of an upstream duplex structure. It also remains to be determined whether RNases H1 cleave dsDNA^R1^ at the (5’)RNA-DNA(3’) junction. We used *E. coli* RNase H1 as a representative member of type 1 RNases H in this study to analyze the 3’-JRNase activity of type 1 RNase H, because the structure and function of *E. coli* RNase H1 have been extensively studied^32^.

In this report, we showed that the RNA-DNA junction of the RNA1-DNA9/DNA18 substrate is not cleaved by *E. coli* RNase H1 but is cleaved by the enzyme when a short RNA fragment is supplied to facilitate the formation of an upstream duplex structure. We also showed that *E. coli* RNase H1 exhibits 3’-JRNase activity for dsDNA^R1^ in the presence of manganese ions much more effectively than in the presence of magnesium ions, regardless of whether this substrate is cleaved by 5’-JRNase activity of *E. coli* RNase H2 in advance or not. From these results, we propose that single rNMPs misincorporated into the genomes can be excised by a cooperative work of RNases H1 and H2 in prokaryotic cells.

## Results and Discussion

### Oligomeric substrates

**Figure 2:**
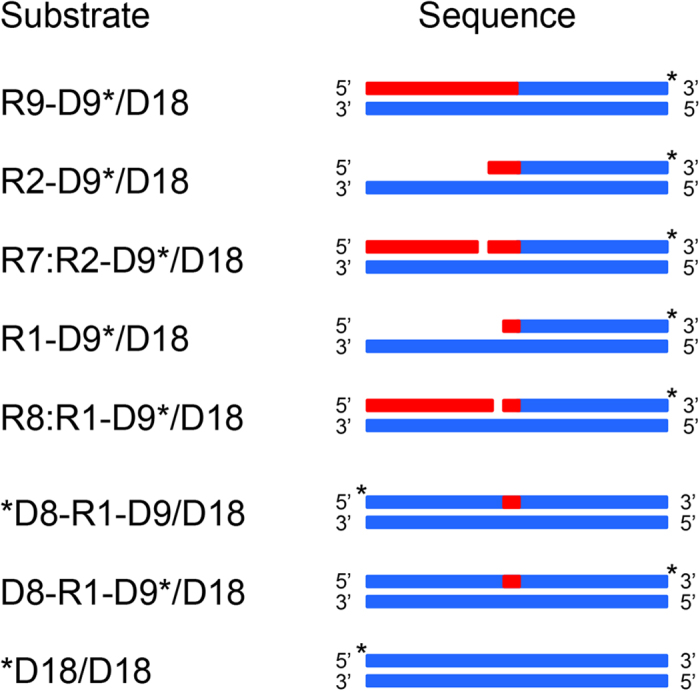
The oligomeric substrates used in this study are schematically shown. The RNA and DNA strands are shown by red and blue boxes respectively. The asterisk indicates the fluorescein-labeled site. The R2-D9* and R1-D9* strands in the R2-D9*/D18, R7:R2-D9*/D18, R1-D9*/D18, and R8:R1-D9*/D18 duplexes are 5’-phosphorylated.

The oligomeric substrates used in this study are summarized in Fig. 2. The asterisk indicates the fluorescein-labeled site. The RNA_9_-DNA_9_*/DNA_18_ (R9-D9*/D18) substrate represents an Okazaki fragment-like substrate labeled at the 3’-end. The RNA_1_-DNA_9_*/DNA_18_ (R1-D9*/D18) and RNA_2_-DNA_9_*/DNA_18_ (R2-D9*/D18) substrates represent Okazaki fragment-like substrates containing one and two ribonucleotides labeled at the 3’-ends. The R8:R1-D9*/D18 and R7:R2-D9*/D18 substrates represent the R9-D9*/D18 substrates with a nick at the P-O3’ bond between the first and second and between the second and third ribonucleotides from the RNA-DNA junction respectively. The *DNA_8_-RNA_1_-DNA_9_/DNA_18_ (*D8-R1-D9/D18) and DNA_8_-RNA_1_-DNA_9_*/DNA_18_ (D8-R1-D9*/D18) substrates represent dsDNA containing a single ribonucleotide (dsDNA^R1^) labeled at the 5’- and 3’-ends respectively. The *DNA_18_/DNA_18_ (*D18/D18) substrate represents dsDNA labeled at the 5’-end. For an Okazaki fragment-like substrate, the first and second ribonucleotides from the RNA-DNA junction are termed R(−1) and R(−2) respectively. Likewise, the first and second deoxyribonucleotides from the RNA-DNA junction are termed D(+1) and D(+2) respectively. The phosphodiester bonds between R(−2) and R(−1), R(−1) and D(+1), and D(+1) and D(+2) are designated as R(−2)-R(−1), RNA-DNA junction, and D(+1)-D(+2) respectively. For dsDNA^R1^, the 5’- and 3’-sides of the ribonucleotide are designated as (5’)DNA-RNA(3’) and (5’)RNA-DNA(3’) junctions respectively.

### Purity of the proteins

**Figure 3:**
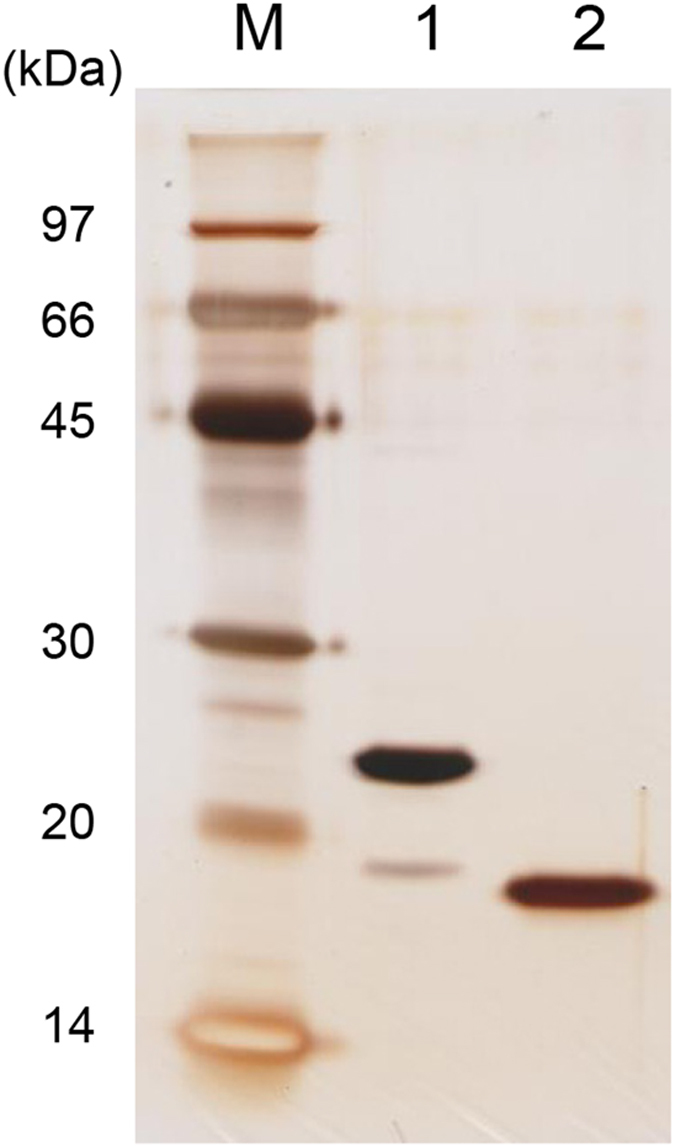
SDS-PAGE of the purified proteins. *E. coli* RNase H2 (lane 1) and *E. coli* RNase H1 (lane 2) were subjected to electrophoresis on a 15% polyacrylamide gel in the presence of SDS. The amount of the protein was 0.5 μg. After electrophoresis, the protein was detected by silver staining of gel. Lane M, a low-molecular-weight marker kit (GE Healthcare).

*E. coli* RNases H1 and H2 are encoded by the *rnhA* and *rnhB* genes respectively. The purity of these proteins used in this study were analyzed by SDS-PAGE, followed by silver staining. The results are shown in Fig. 3. *E. coli* RNase H1 migrates in the gel as a single band, indicating that it is homogeneous. *E. coli* RNase H2 does not migrate in the gel as a single band, but migrates as a major band.

### Cleavage of R9-D9/D18 substrate by E. coli RNase H1

It has been reported that *E. coli* RNase H1 cleaves an Okazaki fragment-like substrate most effectively at R(−2)-R(−1) and less effectively at the RNA-DNA junction in the presence of 5 mM MnCl_2_^31^, indicating that *E. coli* RNase H1 exhibits a weak 3’-JRNase activity for this substrate in the presence of manganese ions. However, two conflicting results that *E. coli* RNase H1 can^26^,^31^ and cannot^29^,^30^ cleave this substrate at the RNA-DNA junction in the presence of magnesium ions have been reported. Therefore, the R9-D9*/D18 substrate was first cleaved by *E. coli* RNase H1 either in the presence of 0.1 mM MnCl_2_ or 10 mM MgCl_2_, to examine whether *E. coli* RNase H1 exhibits 3’-JRNase activity for this substrate in the presence of magnesium ions. The results are shown in Figs. 3a, b. The D9* fragment is detected as one of the major products when the substrate is extensively cleaved by the enzyme. This band is produced more effectively in the presence of manganese ions than in the presence of magnesium ions. These results indicate that *E. coli* RNase H1 cleaves the R9-D9*/D18 substrate at the RNA-DNA junction either in the presence of manganese or magnesium ions, but more effectively in the presence of manganese ions than in the presence of magnesium ions. This result is consistent with that previously reported^31^. Thus, *E. coli* RNase H1 exhibits 3’-JRNase activity for an Okazaki fragment-like substrate either in the presence of manganese or magnesium ions, but more strongly in the presence of manganese ions. This metal ion preference is opposite to that for the RNase H activity of this enzyme determined using an RNA/DNA substrate^33^.

**Figure 4:**
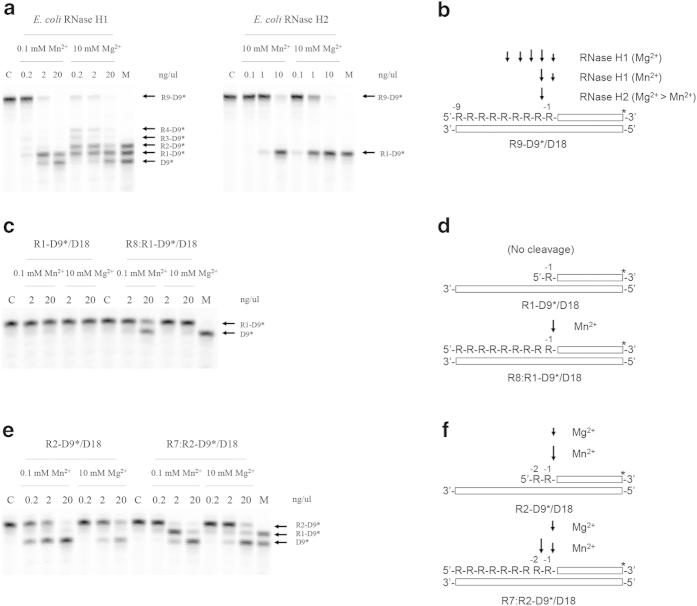
Cleavage of R9-D9/D18, R1-D9/D18, and R2-D9/D18 substrates. (**a**,**c**, and **e**) The R9-D9*/D18 substrate (**a**), R1-D9*/D18 and R8:R1-D9*/D18 substrates (**c**), and R2-D9*/D18 and R7:R2-D9*/D18 substrates (**e**) were hydrolyzed by the enzyme at 30 °C for 15 min and the products were separated on a 20% polyacrylamide gel containing 7 M urea as described in Experimental Procedures. The concentration of the substrate was 1.0 μM. The enzymes used to cleave these substrates are shown above the gel. The concentrations of these enzymes in the reaction mixture (10 μL) are indicated above each lane. The metal cofactors used to cleave these substrates are also shown above the gel together with their concentrations. C, substrate before enzymatic reaction; M, marker. (**b**, **d**, and **f**) The cleavage sites are schematically shown. “R” represents the ribonuclotide, which is inversely numbered from −1 from the RNA-DNA junction to the 5’ end. The open box represents the DNA strand (either D9 or D18). The differences in the lengths of the arrows reflect relative cleavage intensities at the position indicated.

*E. coli* RNase H1 cleaves the R9-D9*/D18 substrate almost exclusively at R(−2)-R(−1) and RNA-DNA junction in the presence of 0.1 mM MnCl_2_, whereas it cleaves this substrate preferentially at R(−3)-R(−2) and R(−2)-R(−1) in the presence of 10 mM MgCl_2_ (Fig. 3a,b). It has been reported that hydrolysis of this substrate by *E. coli* RNase H1 is initiated by the cleavage at R(−5)-R(−4), R(−4)-R(−3), and R(−3)-R(−2) in the presence of 10 mM MgCl_2_^26^. These results suggest that the R9-D9*/D18 substrate is cleaved by *E. coli* RNase H1 preferentially at the upstream region of the RNA-DNA junction in the presence of magnesium ions due to its RNase H activity. This substrate is not cleaved by *E. coli* RNase H1 at the upstream region of the RNA-DNA junction in the presence of 0.1 mM MnCl_2_, except for R(−2)-R(−1), probably due to a very weak RNase H activity. It has been reported that the RNase H activity of *E. coli* RNase H1 determined using an RNA/DNA substrate in the presence of the optimum concentration of manganese ions (2–4 μM) is lower than that determined in the presence of the optimum concentration of magnesium ions (5–10 mM) by only 5 fold^33^, whereas the RNase H activity of *E. coli* RNase H1 determined using a 12 base pair RNA/DNA substrate in the presence of 5 mM MnCl_2_ is lower than that determined in the presence of 5 mM MgCl_2_ by 1000 fold^31^. As a result, *E. coli* RNase H1 cleaves the R9-D9*/D18 substrate more effectively in the presence of 10 mM MgCl_2_ than in the presence of 0.1 mM MnCl_2_ (Fig. 4a).

### Cleavage of R1-D9/D18 and R8:R1-D9/D18 substrates by E. coli RNase H1

The R1-D9* fragment is detected as one of the major products when the R9-D9*/D18 substrate is extensively cleaved by *E. coli* RNase H1 (Fig. 4a), suggesting that the R1-D9*/D18 substrate is not cleaved by *E. coli* RNase H1. To examine whether *E. coli* RNase H1 does not cleave this substrate, but cleaves it at the RNA-DNA junction when 8 b RNA (R8) complementary to the single stranded region of the R1-D9*/D18 substrate is supplied, the R1-D9*/D18 and R8:R1-D9*/D18 substrates were cleaved by *E. coli* RNase H1 either in the presence of 0.1 mM MnCl_2_ or 10 mM MgCl_2_. The results are shown in Figs. 3c, d. *E. coli* RNase H1 does not cleave the R1-D9*/D18 substrate regardless of the metal cofactors. In contrast, it cleaves the R8:R1-D9*/D18 substrate at the RNA-DNA junction, but only in the presence of manganese ions. This result suggests that *E. coli* RNase H1 cleaves an Okazaki fragment-like RNA-DNA/DNA substrate at the RNA-DNA junction regardless of whether this substrate contains a nick at R(−2)-R(−1). The RNA-DNA junction of the R8:R1-D9*/D18 substrate is not fully cleaved by *E. coli* RNase H1, probably because the upstream region of the RNA-DNA junction is cleaved before the RNA-DNA junction is completely cleaved. The RNA-DNA junction of the R9-D9*/D18 substrate is cleaved by the 3’-JRNase activity of *E. coli* RNase H1 less effectively in the presence of magnesium ions than in the presence of manganese ions, probably because the upstream region of the RNA-DNA junction is cleaved by the RNase H activity of *E. coli* RNase H1 more effectively in the presence of magnesium ions than in the presence of manganese ions. The RNA-DNA junction of the R8:R1-D9*/D18 substrate is not cleaved by *E. coli* RNase H1 in the presence of magnesium ions, probably because the presence of a nick at R(−2)-R(−1) alters the interaction between the substrate and metal ion, in such a way that the scissile phosphate group of the substrate and magnesium ions are not arranged ideally.

### Cleavage of R2-D9/D18 and R7:R2-D9/D18 substrates by E. coli RNase H1

Inability of *E. coli* RNase H1 to cleave the R1-D9*/D18 substrate at the RNA-DNA junction indicates that multiple upstream ribonucleotides are necessary for the cleavage of an Okazaki fragment-like substrate by the enzyme at the RNA-DNA junction. To examine whether two upstream ribonucleotides are sufficient for this cleavage, the R2-D9*/D18 substrate was cleaved by *E. coli* RNase H1 either in the presence of 0.1 mM MnCl_2_ or 10 mM MgCl_2_. The results are shown in Figs. 3e, f. *E. coli* RNase H1 cleaves the R2-D9*/D18 substrate at the RNA-DNA junction either in the presence of manganese or magnesium ions, but more effectively in the presence of manganese ions. This result indicates that the presence of two upstream ribonucleotides is sufficient for the cleavage of an Okazaki fragment-like substrate by *E. coli* RNase H1 at the RNA-DNA junction. To examine whether this cleavage site is shifted by supplying an RNA strand that facilitates the formation of an upstream duplex structure, the R7:R2-D9*/D18 substrate was also cleaved by *E. coli* RNase H1. The results are shown in Fig. 3e, f. *E. coli* RNase H1 most effectively cleaves this substrate at R(−2)-R(−1) in the presence of manganese ions. This site is also cleaved in the presence of magnesium ions, but much less effectively, probably because the RNA/DNA region of the R7:R2-D9*/D18 substrate is effectively cleaved by the RNase H activity of the enzyme in the presence of magnesium ions. Thus, the primary products of the R7:R2-D9*/D18 substrate upon cleavage with *E. coli* RNase H1 in the presence of manganese and magnesium ions are probably the R7:R1-D9*/D18 and R2-D9*/D18 duplexes respectively. The RNA-DNA junctions of these primary products are cleaved only when the concentration of the enzyme is greatly elevated.

### Cleavage of D8-R1-D9/D18 substrate by E. coli RNase H1

**Figure 5:**
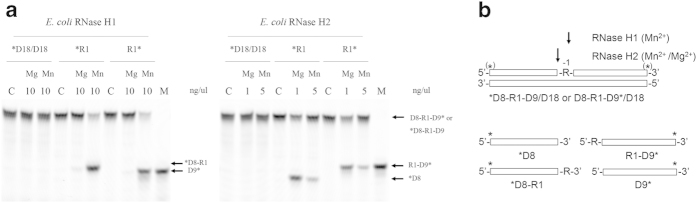
Cleavage of D8-R1-D9/D18 substrate. (**a**) The *D8-R1-D9/D18, D8-R1-D9*/D18, and *D18/D18 substrates (1.0 μM) were hydrolyzed by *E. coli* RNase H1 (10 ng μL^−1^) or *E. coli* RNase H2 (1 ng μL^−1^ or 5 ng μL^−1^) at 30 °C for 15 min in the presence of 10 mM MgCl_2_ or 0.1 mM MnCl_2_ for *E. coli* RNase H1 or in the presence of 10 mM MgCl_2_ or 10 mM MnCl_2_ for *E. coli* RNase H2 and the products were separated on a 20% polyacrylamide gel containing 7 M urea. The enzymes, metal cofactors, and substrates are shown above the gel. *R1 and R1* represent *D8-R1-D9/D18 and D8-R1-D9*/D18 respectively. C, substrate before enzymatic reaction; M, marker. (**b**) The cleavage sites and the products detected by gel electrophoresis are schematically shown. “R” represents the ribonuclotide. The open box represents the DNA strand. The differences in the lengths of the arrows reflect relative cleavage intensities at the position indicated.

To examine whether *E. coli* RNase H1 exhibits 3’-JRNase activity for dsDNA^R1^, the D8-R1-D9*/D18 and *D8-R1-D9/D18 substrates were cleaved by *E. coli* RNase H1 either in the presence of 0.1 mM MnCl_2_ or 10 mM MgCl_2_. The results are shown in Fig. 5. The D9* and *D8-R1 fragments are detected as the major products more clearly in the presence of manganese ions, indicating that *E. coli* RNase H1 cleaves these substrates mainly at the (5’)RNA-DNA(3’) junction and much more effectively in the presence of manganese ions than in the presence of magnesium ions to produce the D8-R1-D9/D18 duplex containing a nick at the (5’)RNA-DNA(3’) junction. The D9* fragment produced from D8-R1-D9* upon cleavage with *E. coli* RNase H1 did not migrate in the urea gel equally with the synthetic D9* fragment having the 5’-OH terminus but migrated equally with the synthetic D9* fragment phosphorylated at the 5’-terminus. The synthetic D9* fragment phosphorylated at the 5’-terminus migrated in the urea gel faster and slower than the synthetic D9* and D8* fragments with the 5’-OH terminus respectively (data not shown). This indicates that the 3’-JRNase activity of *E. coli* RNase H1 hydrolyzes the phosphodiester bond (PO-3’) of the (5’)RNA-DNA(3’) junction producing a 3’-OH and a 5’-phosphate ended products. *E. coli* RNase H1 does not cleave the *D18/D18 substrate at all either in the presence of 0.1 mM MnCl_2_ or 10 mM MgCl_2_, indicating that the presence of the ribonucleotide is required for the cleavage of the D8-R1-D9/D18 substrate by the 3’-JRNase activity of *E. coli* RNase H1. Determination of the 3’-JRNase activity of *E. coli* RNase H1 in the presence of various concentrations (0.1–100 mM) of manganese or magnesium ions using the D8-R1-D9*/D18 substrate indicated that *E. coli* RNase H1 exhibits 3’-JRNase activity most efficiently in the presence of manganese ions and the optimum concentration of manganese ions for this activity is 0.1 mM (data not shown). This result suggests that the substrate specificity of *E. coli* RNase H1 is less specific in the presence of manganese ions than in the presence of magnesium ions. Decreased substrate specificity and cleavage-site selectivity in the presence of manganese ions have also been reported for other RNases H^23^,^24^,^26^,^27^,^34^.

### Cleavage of R9-D9/D18 and D8-R1-D9/D18 substrates by E. coli RNase H2

The finding that *E. coli* RNase H1 can cleave the D8-R1-D9/D18 substrate at the (5’)RNA-DNA(3’) junction promotes us to examine whether a single ribonucleotide of dsDNA^R1^ can be removed by the combination of the 5’-JRNase activity of *E. coli* RNase H2 and the 3’-JRNase activity of *E. coli* RNase H1. *E. coli* RNase H2 has been shown to exhibit 5’-JRNase activity for an Okazaki fragment-like substrate^31^. A crude extract from *E. coli* cells exhibits 5’-JRNase activity for dsDNA^R1^, whereas that from RNase H2-deficient *E. coli* cells does not exhibit this activity^22^, suggesting that *E. coli* RNase H2 also exhibits 5’-JRNase activity for dsDNA^R1^. However, it remains to be determined whether the purified protein of *E. coli* RNase H2 exhibits this activity for dsDNA^R1^. Therefore, the *D8-R1-D9/D18 and D8-R1-D9*/D18 substrates were cleaved by *E. coli* RNase H2 either in the presence of 10 mM MnCl_2_ or 10 mM MgCl_2_, to examine whether this enzyme exhibits 5’-JRNase activity for dsDNA^R1^. The R9-D9*/D18 substrate was also cleaved by this enzyme for comparative purpose either in the presence of 10 mM MnCl_2_ or 10 mM MgCl_2_.

As shown in Fig. 3a, b, *E. coli* RNase H2 cleaved the R9-D9*/D18 substrate almost exclusively at R(−2)-R(−1) either in the presence of manganese or magnesium ions, but more effectively in the presence of magnesium ions. Likewise, as shown in Fig. 5, *E. coli* RNase H2 cleaved the *D8-R1-D9/D18 and D8-R1-D9*/D18 substrates almost exclusively at the (5’)DNA-RNA(3’) junction to produce the D8-R1-D9/D18 duplex containing a nick at the (5’)DNA-RNA(3’) junction either in the presence of manganese or magnesium ions, but more effectively in the presence of magnesium ions. Thus, *E. coli* RNase H2 exhibits 5’-JRNase activity for dsDNA^R1^ either in the presence of manganese or magnesium ions, but more strongly in the presence of magnesium ions. This metal ion preference is opposite to that for the RNase H activity of this enzyme determined using an RNA/DNA substrate^31^,^35^. The RNase H activity of *E. coli* RNase H2 determined in the presence of 5 mM MgCl_2_ is lower than that determined in the presence of 5 mM MnCl_2_ by 10 fold. The RNA/DNA hybrid region of the R9-D9*/D18 substrate is not cleaved by *E. coli* RNase H2, probably because the RNase H activity of this enzyme is very low. It has been reported that the RNase H activity of *E. coli* RNase H2 determined in the presence of 5 mM MnCl_2_ using an oligomeric RNA/DNA substrate is lower than that of *E. coli* RNase H1 determined in the presence of 5 mM MgCl_2_ and 5 mM MnCl_2_ by 2 × 10^4^ and 20 fold respectively^31^. Determination of the 5’-JRNase activity of *E. coli* RNase H2 in the presence of various concentrations (0.1–100 mM) of manganese or magnesium ions using the D8-R1-D9*/D18 substrate indicates that the optimum concentrations of manganese and magnesium ions for this activity are both 10 mM and the activity determined in the presence of 10 mM MgCl_2_ is higher than that determined in the presence of 10 mM MnCl_2_ by 15 fold (data not shown).

### Stepwise cleavage of D8-R1-D9/D18 substrate by E. coli RNases H1 and H2

**Figure 6:**
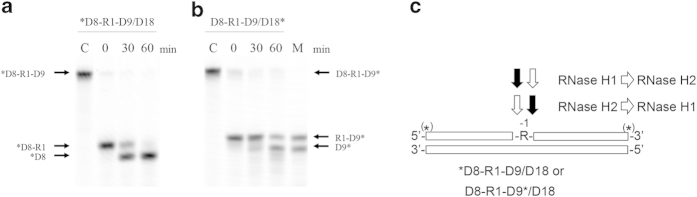
Stepwise cleavage of D8-R1-D9/D18 substrate with *E. coli* RNases H1 and H2. (**a**) The *D8-R1-D9/D18 substrate (1.0 μM) was hydrolyzed by *E. coli* RNase H1 (20 ng μL^−1^) at 30 °C for 30 min in the presence of 0.1 mM MnCl_2_ at first. The resultant product, *D8-R1:D9/D18 duplex, was further hydrolyzed by *E. coli* RNase H2 (10 ng μL^−1^) at 30 °C for the period indicated above each lane in the presence of 0.1 mM MnCl_2_ and 1 mM MgCl_2_ and the products were separated on a 20% polyacrylamide gel containing 7 M urea. *R1 represents *D8-R1-D9/D18. C, substrate before enzymatic reaction. (**b**) The D8-R1-D9*/D18 substrate (1.0 μM) was hydrolyzed by *E. coli* RNase H2 (10 ng μL^−1^) at 30 °C for 30 min in the presence of 1 mM MgCl_2_ at first. The resultant product, D8:R1-D9*/D18 duplex, was further hydrolyzed by *E. coli* RNase H1 (20 ng μL^−1^) at 30 °C for the period indicated above each lane in the presence of 0.1 mM MnCl_2_ and 1 mM MgCl_2_ and the products were separated on a 20% polyacrylamide gel containing 7 M urea. R1* represents D8-R1-D9*/D18. C, substrate before enzymatic reaction; M, markers. (**c**) The cleavage sites are schematically shown. “R” represents the ribonuclotide. The open box represents the DNA strand. The open and solid arrows represent the first and second cleavage sites respectively.

To examine whether a single ribonucleotide embedded in dsDNA can be removed by the combination of the 5’-JRNase activity of *E. coli* RNase H2 and 3’-JRNase activity of *E. coli* RNase H1, the D8-R1-D9/D18 substrate was cleaved by these enzymes in a stepwise manner. The results are shown in Fig. 6. When the *D8-R1-D9/D18 substrate was cleaved by *E. coli* RNase H1 in the presence of 0.1 mM MnCl_2_ in the first step, and by *E. coli* RNases H1 and H2 in the presence of both 0.1 mM MnCl_2_ and 1 mM MgCl_2_ in the second step, the (5’)RNA-DNA(3’) and (5’)DNA-RNA(3’) junctions of the substrate were cleaved in the first and second steps respectively. As a result, the single ribonucleotide was removed from the substrate. Likewise, when the D8-R1-D9*/D18 substrate was incubated with *E. coli* RNase H2 in the presence of 1 mM MgCl_2_ in the first step, and by *E. coli* RNases H1 and H2 in the presence of both 0.1 mM MnCl_2_ and 1 mM MgCl_2_ in the second step, the (5’)DNA-RNA(3’) and (5’)RNA-DNA(3’) junctions of the substrate were cleaved in the first and second steps respectively. As a result, the single ribonucleotide was removed from the substrate. These results suggest that the presence of manganese and magnesium ions is not inhibitory for the 5’-JRNase activity of *E. coli* RNase H2 and 3’-JRNase activity of *E. coli* RNase H1 respectively. These results also suggest that *E. coli* RNases H1 and H2 exhibit 3’- and 5’-JRNase activities for dsDNA^R1^ with a nick at the (5’)DNA-RNA(3’) and (5’)RNA-DNA(3’) junctions respectively.

Alternatively, when examining the 3’-JRNase activity using D15-R1-D13/D29 substrate having an adenosine as the single ribonucleotide instead of cytidine and surrounded by AA on each side instead of TGC in the case of D8-R1-D9/D18 substrate used in this study, *E. coli* RNase H1 cleaves the D15-R1-D13/D29 substrate most effectively at the (5’)RNA-DNA(3’) junction and less effectively at D(+1)-D(+2), whereas it almost exclusively cleaves this substrate at D(+1)-D(+2) when a nick is introduced at the (5’)DNA-RNA(3’) junction (E. Tannous, unpublished results). These contradictory results may be due to the difference in the single ribonucleotide selected and the DNA sequence surrounding it which may alter the interaction between the enzyme and substrate in such a way that the D(+1)-D(+2) bond, instead of (5’)RNA-DNA(3’) junction, contacts the active site of the enzyme.

### Cleavage of D8-R1-D9/D18 substrate by mixture of E. coli RNases H1 and H2

**Figure 7:**
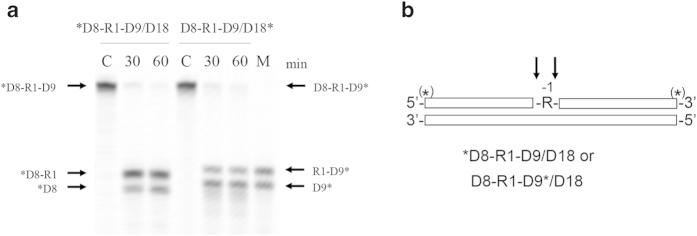
Cleavage of D8-R1-D9/D18 substrate with a mixture of *E. coli* RNases H1 and H2. (**a**) The *D8-R1-D9/D18 or D8-R1-D9*/D18 substrate (1.0 μM) was hydrolyzed by a mixture of *E. coli* RNase H1 (20 ng μL^−1^) and *E. coli* RNase H2 (10 ng μL^−1^) at 30 °C for the period indicated above each lane in the presence of 0.1 mM MnCl_2_ and 1 mM MgCl_2_. The products were separated on a 20% polyacrylamide gel containing 7 M urea. *R1 and R1* represent *D8-R1-D9/D18 and D8-R1-D9*/D18 respectively. C, substrate before enzymatic reaction; M, markers. (**b**) The cleavage sites are schematically shown as in Fig. 5b. The arrows represent the cleavage sites.

To examine whether single ribonucleotides can be removed from dsDNA^R1^ by a cooperative work of *E. coli* RNases H1 and H2, the D8-R1-D9/D18 substrate was cleaved by a mixture of these enzymes. The results are shown in Fig. 7. When the *D8-R1-D9/D18 substrate was cleaved by the mixture of *E. coli* RNase H1 (20 ng μL^−1^) and *E. coli* RNase H2 (10 ng μL^−1^) in the presence of 1 mM MgCl_2_ and 0.1 mM MnCl_2_, the *D8 fragment was detected as the major product. Likewise, when the D8-R1-D9*/D18 substrate was cleaved by the mixture of these enzymes in the same condition, the D9* fragment was detected as the major product. These results suggest that single ribonucleotides embedded in dsDNA can be removed by a cooperative work of *E. coli* RNases H1 and H2.

### Possible involvement of E. coli RNase H1 in DNA repair

DNA (deoxyribonucleic acid) is a universal hereditary material that encodes genetic information essential for the existence of all cellular life and some viruses. It is characterized by its ability to store and transfer information and to self-replicate, which is catalyzed by enzymatic machinery. To keep the integrity of the transferred information, DNA should be kept unmodified. However, DNA is frequently subject to various modifications that can render it unstable if left unrepaired. Of these modifications, the presence of single ribonucleotide monophosphates (rNMPs) misincorporated into the DNA backbone shows both negative and positive consequences on the genome^36^. While the positive roles shown up to now are limited to nascent strand discrimination in mismatch repair^37^,^38^,^39^ and the use of ribonucleotides by polμ in non-homologous end-joining (NHEJ) pathways^40^,^41^,^42^, more studies emphasized the negative roles such as the increase in mutation rate^43^,^44^,^45^, chromosomal abnormality^11^,^12^, mammalian embryonic lethality^11^,^12^, replication fork barrier^18^,^43^,^46^, and autoimmune diseases^13^,^47^. Recent studies indicate that RNase H2 saves the genome by initiating the pathway which removes those intruders and restores the DNA back to its original form with the assistance of several other enzymes^11^,^12^,^18^,^19^,^20^,^38^,^39^.

**Figure 8:**
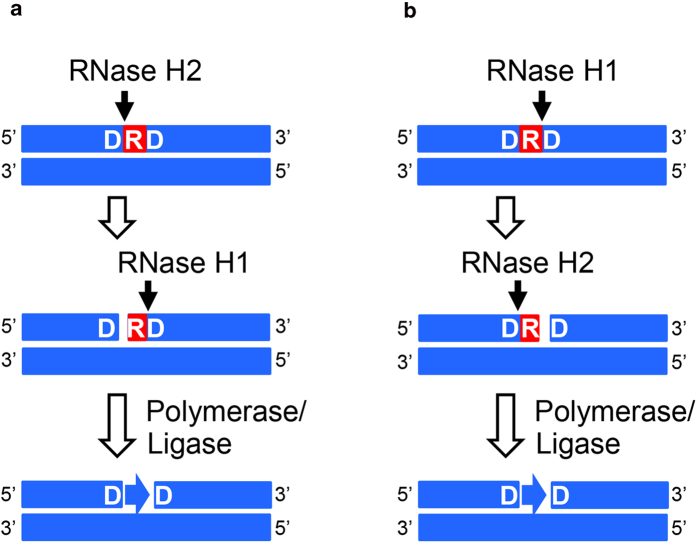
A new RER pathway directed by RNases H1 and H2. dsDNA containing a single ribonucleotide (dsDNA^R1^) is schematically shown. DNA strand and a single ribonucleotide are shown by blue and red boxes respectively. Single ribonucleotide and flanking deoxyribonucleotides are labeled R and D respectively. Two possible pathways, in which a single ribonucleotide embedded in dsDNA is removed by RNases H1 and H2 in a stepwise manner, are shown. In pathway A, dsDNA^R1^ is first cleaved by RNase H2 at the 5’ side of the ribonucleotide to produce dsDNA^R1^ with a nick at the 5’ side of the ribonucleotide. Then dsDNA^R1^ with this nick is subsequently cleaved by RNase H1 at the 3’ side of ribonucleotide of the (5’)RNA-DNA(3’) junction to release a single ribonucleotide. In pathway B, dsDNA^R1^ is first cleaved by RNase H1 at the 3’ side of the ribonucleotide to produce dsDNA^R1^ with a nick at the 3’ side of the ribonucleotide. Then dsDNA^R1^ with this nick is subsequently cleaved by RNase H2 at the 5’ side of the ribonucleotide to release a single ribonucleotide. The gaps in dsDNAs produced by removal of a single ribonucleotide from dsDNA^R1^ in pathway A or B are probably filled by DNA polymerase and ligase.

In this study, we showed for the first time that *E. coli* RNase H1 exhibits 3’-JRNase activity for dsDNA^R1^ much more effectively in the presence of manganese ions than in the presence of magnesium ions, regardless of whether this substrate is cleaved by 5’-JRNase activity of *E. coli* RNase H2 in advance or not, and can excise the single ribonucleotide in collaboration with *E. coli* RNase H2. These results suggest that not only RNase H2 but also RNase H1 is involved in the RER pathway. Two possible RER pathways, in which both enzymes are included, are schematically shown in Fig. 8. According to these pathways, removal of the single ribonucleotides misincorporated into DNA is initiated by the 5’-JRNase activity of RNase H2 and completed by the 3’-JRNase activity of RNase H1, or vice versa. However, human RNase H1 did not exhibit 3’-JRNase activity for dsDNA^R1^ either in the presence of magnesium or manganese ions (E. Tannous, unpublished result), suggesting that RNase H1 is involved in the RER pathway only in the prokaryotic cells. This result and the result that Halo-RNase H1 exhibits 3’-JRNase activity for dsDNA^R1^ in the presence of Mn^2+^ ions (E. Tannous, unpublished result) may exclude the possibility that the 3’-JRNase activity of *E. coli* RNase H1 is derived from another *E. coli* enzyme contaminated, which is not detected as a band on SDS-PAGE (Fig. 4), because human RNase H1 is a basic protein similar to *E. coli* RNase H1 and is purified using cation-exchange column chromatography (E. Tannous, unpublished result), whereas Halo-RNase H1 is an acidic protein and is purified by anion-exchange column chromatography^48^. Because *E. coli* RNase H1 is purified using cation-exchange column chromatography^49^, it is unlikely that another *E. coli* enzyme with 3’-JRNase activity is co-purified with *E. coli* RNase H1 and Halo-RNase H1 but not co-purified with human RNase H1. Disruption of the *rnhA* gene has been reported to increase a basal level of SOS expression in *E. coli*, probably due to persistence of R-loops on the chromosome^50^. However, the 3’-JRNase activity of *E. coli* RNase H1 may not be involved in SOS response, because this activity may not be required for R-loop resolution.

The concentration of magnesium ions (10 mM) optimum for *in vitro* 5’-JRNase activity of *E. coli* RNase H2 is comparable to that in *E. coli* cells, whereas the concentration of manganese ions (0.1 mM) optimum for *in vitro* 3’-JRNase activity of *E. coli* RNase H1 is higher than that in *E. coli* cells. However, the fact that the presence of magnesium ions is not inhibitory for the 3’-JRNase activity of *E. coli* RNase H1 may suggest that *E. coli* RNase H1 exhibits this activity inside the cells. The *in vivo* studies showing that *E. coli* RNase H1 helps in sanitizing the genome from errant rNMPs misincorporated in dsDNA when the *rnhB* gene encoding *E. coli* RNase H2 is inactivated and thus limiting the consequences of the excessive accumulation of ribonucleotides in the *E. coli* genome^20^ supports this hypothesis. Thus, the significance of this work is that it highlights a new possible role for bacterial type 1 RNases H in DNA repair, which should be further investigated *in vivo*, as well as for RNases H from different organisms.

## Methods

### Protein preparation

Overproduction and purification of *E. coli* RNase H1 using *E. coli* MIC3009 transformants with pJAL600^49^ and *E. coli* RNase H2 using *E. coli* MIC2067(DE3) transformants with pTYB600E^35^ were performed as described previously. The purity of each protein was analyzed by SDS-PAGE^51^ using 15% (w/v) polyacrylamide gel, followed by staining with silver staining, using Silver Stain II Kit *Wako* (Wako Pure Chemical Industries, Ltd., Osaka, Japan). The protein concentration was determined from UV absorption using a cell with an optical path length of 1 cm and an *A*_*280*_ value for 0.1% solution of 2.02 for *E. coli* RNase H1 and 0.56 for *E. coli* RNase H2. The value of *E. coli* RNase H1 was experimentally determined^52^. The value of *E. coli* RNase H2 was calculated by using absorption coefficients of 1576 M^−1^ cm^−1^ for Tyr and 5225 M^−1^ cm^−1^ for Trp at 280 nm^53^.

### Oligonucleotides

3’-FAM-labeled 18 b RNA_9_-DNA_9_ (R9-D9*), 11 b RNA_2_-DNA_9_ (R2-D9*), and 10 b RNA_1_-DNA_9_ (R1-D9*), 5’- and 3’-FAM-labeled 18 b DNA_8_-RNA_1_-DNA_9_ (*D8-R1-D9 and D8-R1-D9* respectively), 5’-FAM-labeled 18 b DNA (*D18), 7 b RNA (R7), 8 b RNA (R8), and 18 b DNA (D18) were synthesized by Hokkaido System Science (Sapporo, Japan). Of these oligonucleotides, R2-D9* and R1-D9* are 5’-phosphorylated, while the others are not. The sequences of these oligonucleotides are 5’-uugcaugccTGCAGGTCG-3’ for R9-D9*, 5’-ccTGCAGGTCG-3’ for R2-D9*, 5’-cTGCAGGTCG-3’ for R1-D9*, 5’-uugcaug-3’ for R7, 5’-uugcaugc-3’ for R8, and 5’-TTGCATGCcTGCAGGTCG-3’ for *D8-R1-D9 and D8-R1-D9*, where deoxyribonucleotides and ribonucleotides are shown by uppercase and lowercase letters respectively. The sequence of *D18 is identical to that of *D8-R1-D9. The sequence of D18 is complementary to those of R9-D9* and *D8-R1-D9. FAM represents 6-carboxyfluorescein.

### Enzymatic activity

The R9-D9*/D18, R2-D9*/D18, R1-D9*/D18, *D8-R1-D9/D18, D8-R1-D9*/D18, and *D18/D18 duplexes were prepared by hybridizing R9-D9*, R2-D9*, R1-D9*, *D8-R1-D9, D8-R1-D9*, and *D18 with a 2 molar equivalent of D18 respectively. The R7:R2-D9*/D18 and R8:R1-D9*/D18 duplexes were prepared by hybridizing R2-D9* and R1-D9* with a 2 molar equivalent of D18 in the presence of a 2 molar equivalent of R7 and R8 respectively. These duplexes were used as substrates. Hydrolysis of the substrate at 30 °C for 15 min and separation of the products on a 20% polyacrylamide gel containing 7 M urea were carried out as described previously^54^. The reaction buffer contained 10 mM Tris-HCl (pH 8.5), 1 mM 2-mercaptoethanol, 0.01% BSA, 10 mM NaCl, and MnCl_2_ or MgCl_2_ at the concentration indicated. The substrate concentration was 1 μM. The products were detected by Typhoon 9240 Imager (GE Healthcare, Tokyo, Japan).

## Author Contributions

Conception and design of the work and final approval of the version to be published: S.K. Acquisition and analysis of data and preparation of the paper: E.T. Interpretation and discussion of data: E.T., E.K. and S.K.

## Additional Information

**How to cite this article**: Tannous, E. *et al.* Role of RNase H1 in DNA repair: removal of single ribonucleotide misincorporated into DNA in collaboration with RNase H2. *Sci. Rep.* **5**, 9969; doi: 10.1038/srep09969 (2015).

## Figures and Tables

**Figure 1:**
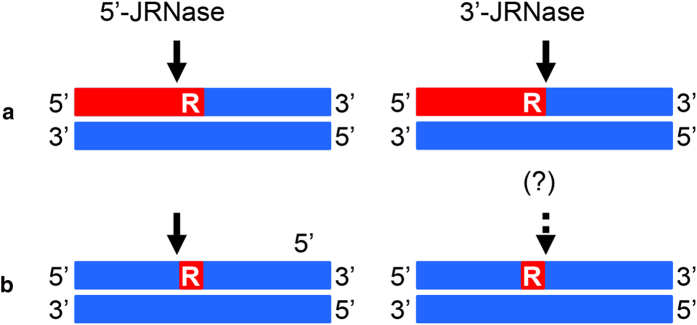
Sites of cleavage by 5’- and 3’-JRNase activities of RNases H. Cleavage sites of an Okazaki fragment-like RNA-DNA/DNA substrate (**a**) and dsDNA containing a single ribonucleotide (dsDNA^R1^) (**b**) by 5’- and 3’-JRNase activities of RNases H are shown. RNA and DNA strands are shown by red and blue boxes respectively. The ribonucleotide of the (5’)RNA-DNA(3’) junction is indicated by R. The cleavage sites are indicated by arrows.
